# Aqueous extracts of Aconite promote thermogenesis in rats with hypothermia via regulating gut microbiota and bile acid metabolism

**DOI:** 10.1186/s13020-021-00437-y

**Published:** 2021-03-19

**Authors:** Juan Liu, Yuzhu Tan, Hui Ao, Wuwen Feng, Cheng Peng

**Affiliations:** 1 State Key Laboratory of Southwestern Chinese Medicine Resources, School of Pharmacy, Chengdu University of Traditional Chinese Medicine, Chengdu, 611130 China; 2 National Key Laboratory Breeding Base of Systematic Research, School of Pharmacy, Chengdu University of Traditional Chinese Medicine, Chengdu, 611130 China

**Keywords:** Aconite, Thermogenesis, Hypothermia, Gut microbiota, Bile acid metabolism

## Abstract

**Background:**

Intermittent or prolonged exposure to severe cold stress disturbs energy homeostasis and can lead to hypothermia, heart failure, Alzheimer’s disease, and so on. As the typical “hot” traditional Chinese medicine, Aconite has been widely used to treat cold-associated diseases for thousands of years, but its critical mechanisms for the promotion of thermogenesis are not fully resolved. Gut microbiota and its metabolites play a crucial role in maintaining energy homeostasis. Here, we investigated whether the aqueous extracts of Aconite (AA) can enhance thermogenesis through modulation of the composition and metabolism of gut microbiota in hypothermic rats.

**Methods:**

The therapeutic effects of AA on body temperature, energy intake, and the histopathology of white adipose tissue and brown adipose tissue of hypothermic rats were assessed. Microbiota analysis based on 16 S rRNA and targeted metabolomics for bile acids (BAs) were used to evaluate the composition of gut microbiota and BAs pool. The antibiotic cocktail treatment was adopted to further confirm the relationship between the gut microbiota and the thermogenesis-promoting effects of AA.

**Results:**

Our results showed a sharp drop in rectal temperature and body surface temperature in hypothermic rats. Administration of AA can significantly increase core body temperature, surface body temperature, energy intake, browning of white adipose tissue, and thermogenesis of brown adipose tissue. Importantly, these ameliorative effects of AA were accompanied by the shift of the disturbed composition of gut microbiota toward a healthier profile and the increased levels of BAs. In addition, the depletion of gut microbiota and the reduction of BAs caused by antibiotic cocktails reduced the thermogenesis-promoting effect of AA.

**Conclusions:**

Our results demonstrated that AA promoted thermogenesis in rats with hypothermia via regulating gut microbiota and BAs metabolism. Our findings can also provide a novel solution for the treatment of thermogenesis-associated diseases such as rheumatoid arthritis, obesity, and type 2 diabetes.

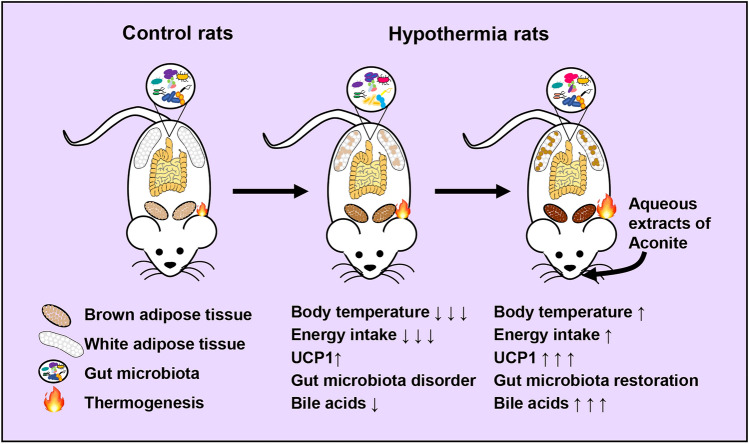

## Background

Adaptive thermogenesis, a basic physiological function obtained from the long history of evolution, plays crucial roles in helping the living organism adapt to a wide range of environmental conditions [1]. When a mammal species is temporarily exposed to a cold environment, some cold receptors sense this stimulation and send the messages to the hypothalamus, resulting in a physiological increase of metabolic rates and shivering thermogenesis to keep the core body temperature [2]. On the contrary, intermittent or prolonged exposure to severe cold stress, a condition that the heat produced cannot compensate for the heat released into the environment, disturbs the homeostasis, resulting in pathological insufficiency of energy metabolism that can be manifested as hypothermia [3]. In addition to hypothermia, clinical and animal studies have demonstrated that exposure to severe cold stress can lead to increased risks for other diseases such as heart failure, Alzheimer’s disease, cancer, musculoskeletal disorders, and rheumatoid arthritis [4–8]. In traditional Chinese medicine (TCM), these diseases induced by cold exposure are classified as cold syndrome-related diseases. Because intermittent or prolonged cold exposure disturbs the energy metabolism of mammals, improving thermogenesis is regarded as an important approach for the treatment of cold associated diseases. At present, the major approach to maintaining body temperature in a cold environment depends on protective measures, such as adding clothing or other items [9, 10]. However, these physical methods only help reduce heat dissipation and cannot restore the homeostasis of energy metabolism.

According to the theory of cold and hot properties of TCM, the TCM with hot property has been widely adopted in China and the surrounding areas for the treatment of cold-related diseases [11]. Aconite (the lateral roots of *Aconitum carmichaelii* Debx.) is the typical “hot” herb that has been extensively used in TCM to treat cold-induced diseases including hypothermia, musculoskeletal disorders, rheumatoid arthritis, etc., for thousands of years [12]. The pharmacologic activities of Aconite include, but are not limited to, cardiovascular system protection, anti-inflammation, and analgesic action [12]. Notably, recent studies have shown that Aconite exhibits strong effects on promoting thermogenesis to prevent cold-stress-induced hypothermia [13]. However, the possible mechanism for the thermogenesis-promoting effects of Aconite remains not fully understood.

In recent years, the gut microbiota has emerged as an important frontier to understand the development of diseases and a burgeoning area to understand the mechanisms of drugs [14–17]. A growing body of evidence has demonstrated that the gut microbiota contributes to energy homeostasis, and its functions are closely associated with thermogenesis [18–20]. Cold exposure can result in a remarkable change of gut microbiota composition, and cold microbiota transplantation to germ-free mice induces the expression of the gene coding for uncoupling protein 1 (UCP1) in brown adipose tissue (BAT) and the browning of inguinal and perigonadal white adipose tissue (WAT), together with increased thermogenesis [21]. It is reported that cold exposure can also increase the levels of bile acids (BAs) in plasma and fecal excretion [22]. BAs play a key functional role in the regulation of thermogenesis. The primary BAs (PBAs) such as cholic acid (CA) and secondary BAs (SBAs, metabolites derived from gut microbiota) such as deoxycholic acid (DCA) can increase the expression of UCP1 in WAT and BAT, and promote WAT browning and BAT thermogenesis [23, 24]. In rodents, browning of WAT and adaptive thermogenesis of BAT are the major contributors to the enhancement of energy metabolism [25]. Taken together, changes in gut microbiota composition and BAs metabolism can regulate the thermogenesis via acting on WAT and BAT. Correspondingly, the improvement of thermogenesis can be achieved through the regulation of gut microbiota and BAs metabolism [26, 27].

Because of the importance of gut microbiota and BA metabolism in thermogenesis, we hypothesize that the aqueous extracts of Aconite (AA) can promote thermogenesis through the remodeling of gut microbiota composition and BA metabolism. In the present study, oral gavage of AA promoted thermogenesis, restored gut microbiota, and increased BAs. Moreover, we found that depletion of gut microbiota weakened the effects of AA on thermogenesis and BAs metabolism. Our study demonstrated the thermogenesis-promoting effects of AA are at least partly through regulating the gut microbiota and BA metabolism. Our results indicated that AA can potentially be used as modulators of the gut microbiota and BAs to promote thermogenesis, thereby combating hypothermia and other cold exposure-related diseases.

## Methods

### Preparation of AA

Aconite (the lateral roots of *Aconitum carmichaelii* Debx., Lot: 180,902) was purchased from Sichuan Jiangyou Zhongba AA Technology Development Co., Ltd. (Jiangyou, China). Aconite slice was weighted and immersed in a 10-fold amount of water (1:10, w/v) for 30 min, and decocted for 5 h. After filtration, an 8-fold mass of water was added and decocted for 3 h and filtered. The filtrate was mixed and concentrated to a dose of 6.25 g crude drug/kg body weight for analysis and administration [28]. Then, it was qualitatively and quantitatively analyzed by ultra-high-performance liquid chromatography coupled with quadrupole time-of-flight mass spectrometry (UPLC-QTOF/MS) and high-performance liquid chromatography (HPLC), respectively.

### Qualitative and quantitative analysis of AA

To qualitatively analyze AA, 100 µL of AA solution was added with 1 mL of methanol. The sample was vortexed for 15 s and centrifuged at 12,000 rpm for 10 min at 4 °C. The supernatant was used for UPLC-QTOF/MS analysis. The chromatographic separation was performed on an HSS T3 column (1.8 μm, 100 mm ⋅ 2.1 mm i.d, Waters Corp., Milford, USA), using solvent A (0.1% formic acid-water) and solvent B (0.1% formic acid-acetonitrile) as the mobile phase for gradient elution. The column temperature was 40 °C, the flow rate was 500 µL/min, and the injection volume was 2 µL. The gradient elution was optimized as 99%−0% A for 16 min, 0% A for 2 min, 0−99% A for 0.1 min, and 99% A for 2 min. The Q-TOF mass spectrometer was operated with a heated electrospray ionization (HESI-II) probe. The positive and negative HESI-II spray voltages were 3 kV and 2.2 kV, respectively, and the ion source temperature was 100 °C. The positive and negative taper hole voltages were 35 v and 30 v, respectively, and the positive and negative heated vaporizer temperatures were 400 °C and 350 °C, respectively. Analyses were made in the full scan mode, and the positive and negative of mass range scanned were 50−1200 *m/z* and 100−1200 *m/z*, respectively. Data were collected and processed with Masslynx V4.1 software (Waters Corp., Milford, USA). Given that the major bioactive components of aconite were alkaloids, we quantitatively analyzed the main alkaloids in the AA. The method for quantitative analysis of alkaloids in AA was the same as our previous study [29].

### Animals, model induction, and drug administration

Specific-pathogen-free (SPF)-grade adult male Wistar rats (280–300 g) were purchased from Chengdu Dossy Experimental Animal Co., Ltd. (Chengdu, China). All animal experiments were approved by the Ethics Committee of Chengdu University of Traditional Chinese Medicine. Rats were housed under a standard environment with a temperature of 22 ± 2 °C, a relative humidity of 60 ± 5%, and a cycle of 12 h light/dark.

After one week acclimatization, 24 rats were randomly allotted to cold water group (n = 12 rats/group) and control group (n = 12 rats/group). The cold water group was subjected to cold water swimming (3.5 ± 0.5 °C, 8 min) for 14 days, and the control group was subjected to warm water swimming (32 ± 0.5 °C, 8 min) for the same days [3, 6]. To rule out the effects of swimming, the control group on the 14th day was randomly divided into the control + cold group (n = 6 rats/group) and control group (n = 6 rats/group). The control + cold group was put into cold water, while the control group was subjected to warm water. Meanwhile, the cold water group on the 14th day was randomly divided into the CH group (n = 6 rats/group) and CH + AA group (n = 6 rats/group). The rats in the CH + AA group were treated with AA (6.25 g crude drug/kg body weight) once a day by gavage, and rats in other groups were given sterile water for 7 days in the same manner. Throughout the trial, body temperature (rectal, eye, dorsal, and ventral region), body weight, and food intake were recorded daily, and feces were collected daily. Before the end of the experiment, feces were collected for the analysis of gut microbiota. At the end of the experiment, serum, inguinal WAT, and interscapular BAT were collected for subsequent analysis. The schematic diagram was shown in Additional file 1: Fig. S1A as experiment 1.

### Antibiotic treatment

After a week of adaptation, 12 rats were randomly divided into CH group (n = 6 rats/group) and CH + ABX group (n = 6 rats/group). Rats in the CH group and CH + ABX group were given sterile water and water with ABX for 14 days, respectively. ABX is consisted of ampicillin (1 g/L), imipenem (250 mg/L), ciprofloxacin (20 mg/L), vancomycin (500 mg/L), and metronidazole (1 g/L) [30]. Then, the CH group was treated with AA (CH + AA) once a day by gavage for 7 days and CH + ABX group was given AA (CH + AA + ABX) in the same way. During the experiment, rats in both groups were given cold-water swimming (3.5 ± 0.5 °C 8 min) once daily. Body weight, body temperature (rectal, eye, dorsal, and ventral region), energy uptake, feces, serum, WAT, and BAT collection and analysis were performed in accordance with experimental 1. The schematic diagram was shown in Additional file 1: Fig. S1B as experiment 2.

### Body temperature measurement

Body temperature (rectal, eye, dorsal, and ventral region) were measured instantly after the rats were removed from the water. Rectal temperature was read with a digital electronic thermometer (TES 1300, TES Electrical Electronic Corp., Taipei, Taiwan). Surface body temperature (eye, dorsal, and ventral region) was recorded with an infrared camera (FLIR ONE ANDROID, UK) from a distance of 20 cm, and the data were further analyzed according to infrared images by FLIR Tools software.

### Energy uptake measurement

During the last three days of the experiment, excreted feces were collected per 24 h. The food and feces were ground into fine powder after drying for 48 h at 60 °C, and then their gross energies were measured with an oxygen bomb calorimeter (Parr, 6100, USA) [31]. Energy uptake was calculated by subtracting the gross energy of the feces from the gross energy in consumed food per 24 h.

### Histology and immunohistochemistry

WAT and BAT were fixed in 4% paraformaldehyde, subsequently embedded in paraffin, and sectioned at 4 μm. Hematoxylin-eosin (H&E) staining was performed according to a standard protocol. The expressions of UCP1 (rabbit anti-UCP1, 23673-1-AP, Proteintech Group, Inc., Wuhan, China) in WAT and BAT were analyzed by immunohistochemistry as the manufacturer’s instructions. The adipocyte size of WAT and expressions of UCP1 were analyzed using Image-Pro Plus 6.0 software (Media Cybernetics Inc., Bethesda, MD, USA).

### 16 S rRNA sequencing and data analysis

Fecal pellets were instantly frozen in liquid nitrogen after collection and stored at − 80 °C. The DNAs were extracted using the E.Z.N.A.® soil kit (Omega Bio-tek, Norcross, GA, U.S.). The V3–V4 region of the 16S rRNA gene of each fecal sample was amplified by the 338F_806R primer pair (the forward primer was 338F: 5′-ACTCCTACGGGAGGCAGCAG-3′ and the reverse primer was 806R: 5′-GGACTACHVGGGTWTCTAAT-3′). The amplification system includes 20 µL FastPfu buffer, 2 µL dNTPs (2.5 nM), 0.8 µL primers (5 µM), and 10 ng of template DNA. Amplification was performed by an initial step at 95 °C for 3 min, followed by 27 cycles at 95 °C for 30 s, 55 °C for 30 s, and 72 °C for 30 s, and a final extension at 72 °C for 10 min. Sequencing was carried out on an Illumina MiSeq PE300 platform (Illumina, San Diego, USA) at Shanghai Majorbio Bio-pharm Technology Co., Ltd (China).

Analysis of gut microbiota composition was performed on MAJORBI CLOUD Platform () based on operational taxonomic units (OTUs), Simpson index, principal component analysis (PCA), and linear discriminate analysis effect size (LEfSe). Phylogenetic investigation of communities by reconstruction of unobserved states (PICRUSt) based on the sequencing of 16 S rRNA gene. The database of Cluster of Orthologous Groups of proteins (COG), Kyoto Encyclopedia of Genes and Genomes (KEGG) as well as KEGG Orthology (KO) were used for the predictive analysis of microbial community function.

### Measurement of BA profiles

A total of 100 µL of serum sample was added with 400 µL of extracting solution (methyl alcohol). The samples were vortexed for 30 s, ultrasonicated for 30 min (5 °C and 40 kHz), stored at −20 °C for 30 min, and centrifuged at 13,000 rpm for 15 min at 4 °C. The supernatant was transferred to a 200 µL vial for ultra-performance liquid chromatography-quadrupole linear ion trap mass spectrometry (UPLC-QTrap-MS/MS) analysis. The chromatographic separation of BAs was performed on a BEH C18 column (1.7 μm, 150 mm ⋅ 2.1 mm i.d, Waters Corp., Milford, USA), using solvent A (0.01% formic acid-water) and solvent B (0.01% formic acid-acetonitrile) as the mobile phase for gradient elution. The column temperature was 45 °C, the flow rate was 300 µL/min, and the injection volume was 2 µL. The gradient elution was optimized as 75%−68% A for 10 min, 68%−25% A for 16 min, 25%−0% A for 0.1 min, 0% A for 2 min, 0%−75% A for 0.1 min, and 75% A for 4 min.

The mass spectrum data were collected by Thermo Q Exactive mass spectrometer. The negative spray voltage was 2.8 kV, and the heated capillary temperature was 320 °C. Both the sheath gas and the auxiliary gas were nitrogen. The flow rates of sheath gas and the auxiliary gas were 40 arb and 10 arb, respectively. The auxiliary gas temperature was 400 °C. The parameters of the mass scan were as follows: the m/z range of 50−750, a full MS resolution of 70,000, and the MS/MS resolution of 17,500. The calibration was customized to keep the mass tolerance of 5 ppm. Data were collected and processed with Xcalibur software (Thermo Scientific Fisher, Boston, USA).

### Statistical analysis

Data were expressed as mean ± standard deviation (*SD*). Statistics were performed using GraphPad Prism version 7 (GraphPad Software Inc, La Jolla, CA, USA). The significance was indicated by one-way analysis of variance (ANOVA) with a post hoc Student-Newman-Keuls test or unpaired two-tailed Student’s *t*-test. Differences with a *P*−values < 0.05 were deemed to be statistically significant, with significance levels marked as *P < 0.05, **P < 0.01, and ***P < 0.001. The significance of bacterial community difference analyses and LEfSe multi-level species discriminant analysis was detected with a non-parametric factorial Kruskal-Wallis sum-rank test. The linear discriminant analysis (LDA) based on the LEfSe method [32] was used to show differences in microbial communities assessed by an LDA score of ≥ 2.

## Results

### Qualitative and quantitative determination of AA

A total of 31 constituents were chemically characterized from the AA (Additional file 1: Table S1) by UPLC-QTOF/MS analysis. The total ion flow diagrams in positive and negative modes were shown in Additional file 1: Fig. S1C and D, respectively. Since the qualitative analysis of AA showed that most of the components were alkaloids, and the major bioactive components of aconite were alkaloids [29], we further quantitatively analyzed the main alkaloids in the AA. The contents of major alkaloids in AA (compared to the weight of crude Aconite) were hypaconitine (0.83 mg/kg), mesaconitine (0.17 mg/kg), benzoylmesaconine (146.90 mg/kg), benzoylhypaconine (74.13 mg/kg), and benzoylaconine (25.56 mg/kg).

### AA improves hypothermia and increases energy intake

**Fig. 1:**
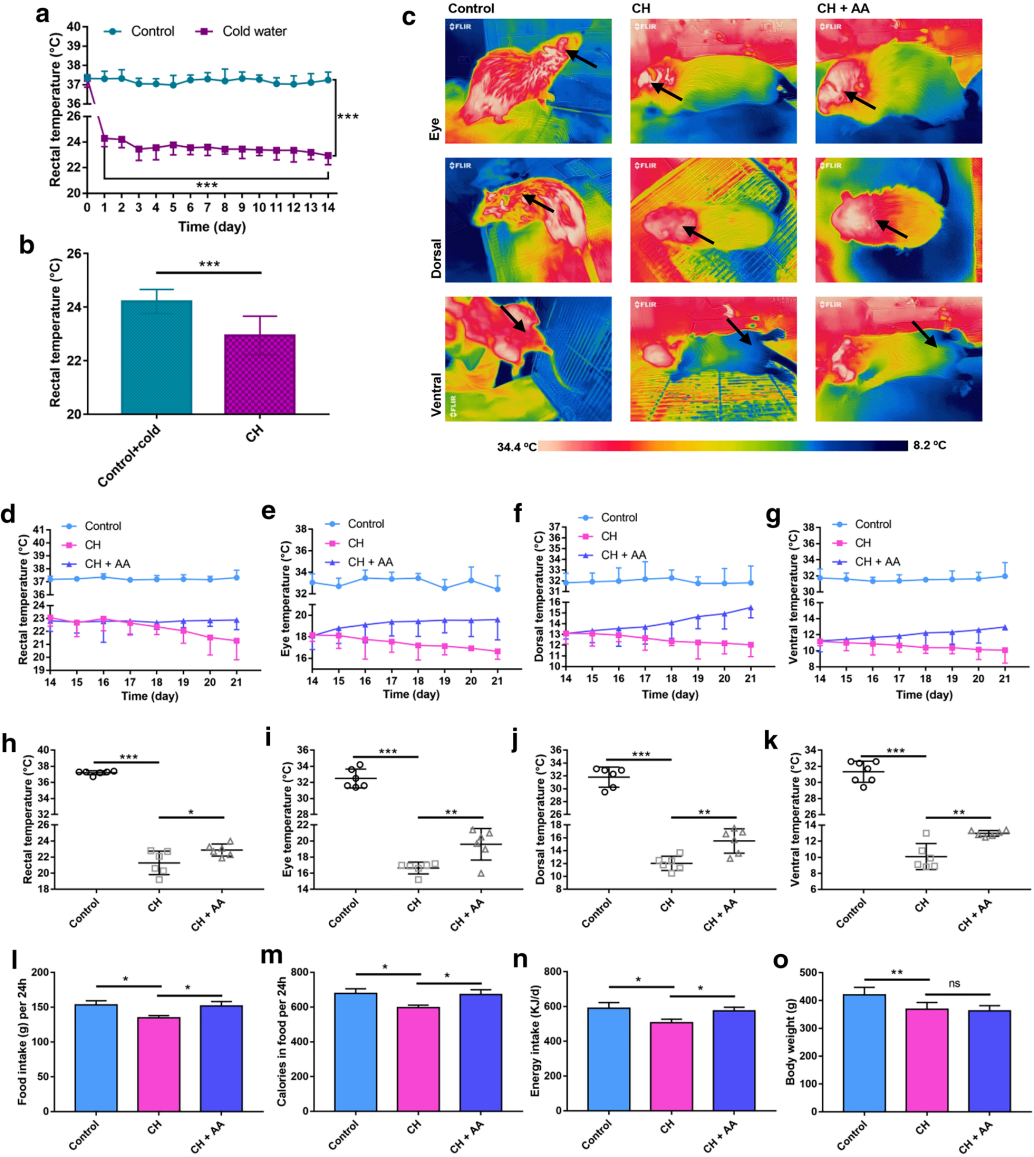
AA increased body temperature and energy intake. **a** Rectal temperature of control group and cold water group from 0 to 14th day. **b** Rectal temperature of control + cold group and CH group on the14th day. **c** Representative infrared images of rats in control group, CH group, and CH + AA group before sacrifice. The black arrow points to the temperature measurement location, including the eyes, back, and abdomen. Temperature trends during AA treatment, including **d** rectal temperature and surface body temperature including eye temperature (**e**), dorsal temperature (**f**), and ventral temperature (**g**).  Rectal temperature (**h**), eye temperature (**i**), dorsal temperature (**j**), and ventral temperature (**k**) on the 21st day. (l–n) Average food intake (**l**), calories in consumed food (**m**), and energy intake (**n**) per 24 h in the last three days. **o** Body weight on the 21st day. Control, CH, and CH + AA represent groups intermittently exposed to warm water (32 ± 0.5 °C), cold water (3.5 ± 0.5 °C), and cold water plus AA, respectively. Data were expressed as mean ± standard deviation (*SD*) (n = 6 rats/group). Differences were evaluated by unpaired two-tailed Student’s *t*-test or one-way ANOVA with a post hoc Student–Newman–Keuls test (*P < 0.05, **P < 0.01, ***P < 0.001, and ns represents no significant difference)

To investigate the effects of severe cold exposure, we monitored the body temperature including rectal temperature (core body temperature) and eye temperature, as well as ventral and dorsal temperature (indicative of the temperature transmitted from WAT and BAT). After 14 days of intermittent exposure to severe cold, the rectal temperature of the cold water group was significantly lower than that of the control group (Fig. 1a). During cold exposure, the rectal temperature of rats was found to decrease gradually (Fig. 1a), which is consistent with the previous report [13]. Moreover, the rectal temperature of the control + cold group was significantly higher than that of the CH group on the 14th day (Fig. 1b), which reflects that the drop in body temperature was caused by cold exposure and was not affected by swimming. These results indicated that intermittent exposure to severe cold pathologically reduced the core body temperature of rats.

To further investigate the effects of severe cold exposure, we measured the temperature of different surface body sites via quantitative infrared imaging (Fig. 1c). Similar to the results of rectal temperature, intermittent exposure to severe cold significantly decreased surface body temperature including eye temperature, dorsal, and ventral temperature on the 14th day (Additional file 1: Fig. S2A−C). The surface body temperature of the cold water group on the 14th day was also significantly lower than that of the same group on the 1st day (Additional file 1: Fig. S2A−C). Furthermore, the surface body temperature of the control + cold group was significantly higher than that of the CH group on the 14th day (Additional file 1: Fig. S2D). These results indicated that intermittent exposure to severe cold pathologically reduced the surface body temperature of rats. Taken together, intermittent exposure to severe cold in rats induced hypothermia, which is manifested as a significant decrease in core body temperature and surface body temperature.

We then treated hypothermic rats with AA to investigate the thermogenesis-promoting effects of AA. Successive administration of AA for 7 days reversed the decreasing trend of body temperature in hypothermic rats (Fig. 1d−g). After 7 days of AA treatment, a significant increase in core body temperature and surface body temperature was observed in the CH + AA group compared with the CH group (Fig. 1h−k). The results indicated that AA treatment improved hypothermia. In addition to body temperature, AA treatment increased the food intake and feces amount (Fig. 1l and Additional file 1: Fig. S2E). To further detect the effects of AA on energy uptake, we measured the food and fecal calories using bomb calorimetry (Fig. 1 m and Additional file 1: Fig. S2F), and calculated total energy intake. As expected, we found a significant reduction in energy intake in the CH rats, and the energy intake was significantly increased by AA treatment (Fig. 1n). Consistent with other studies, intermittent exposure to severe cold decreased body weight in our study (Additional file 1: Fig. S2G) [13, 33]. However, AA treatment did not increase the body weight of rats with hypothermia (Fig. 1o). The results that AA increased energy intake but did not significantly influence body weight of hypothermic rats suggested that AA improved thermogenesis in rats with hypothermia. Taken together, the results showed that AA can improve hypothermia and increase energy intake.

### AA promotes browning of WAT and thermogenesis of BAT

**Fig. 2:**
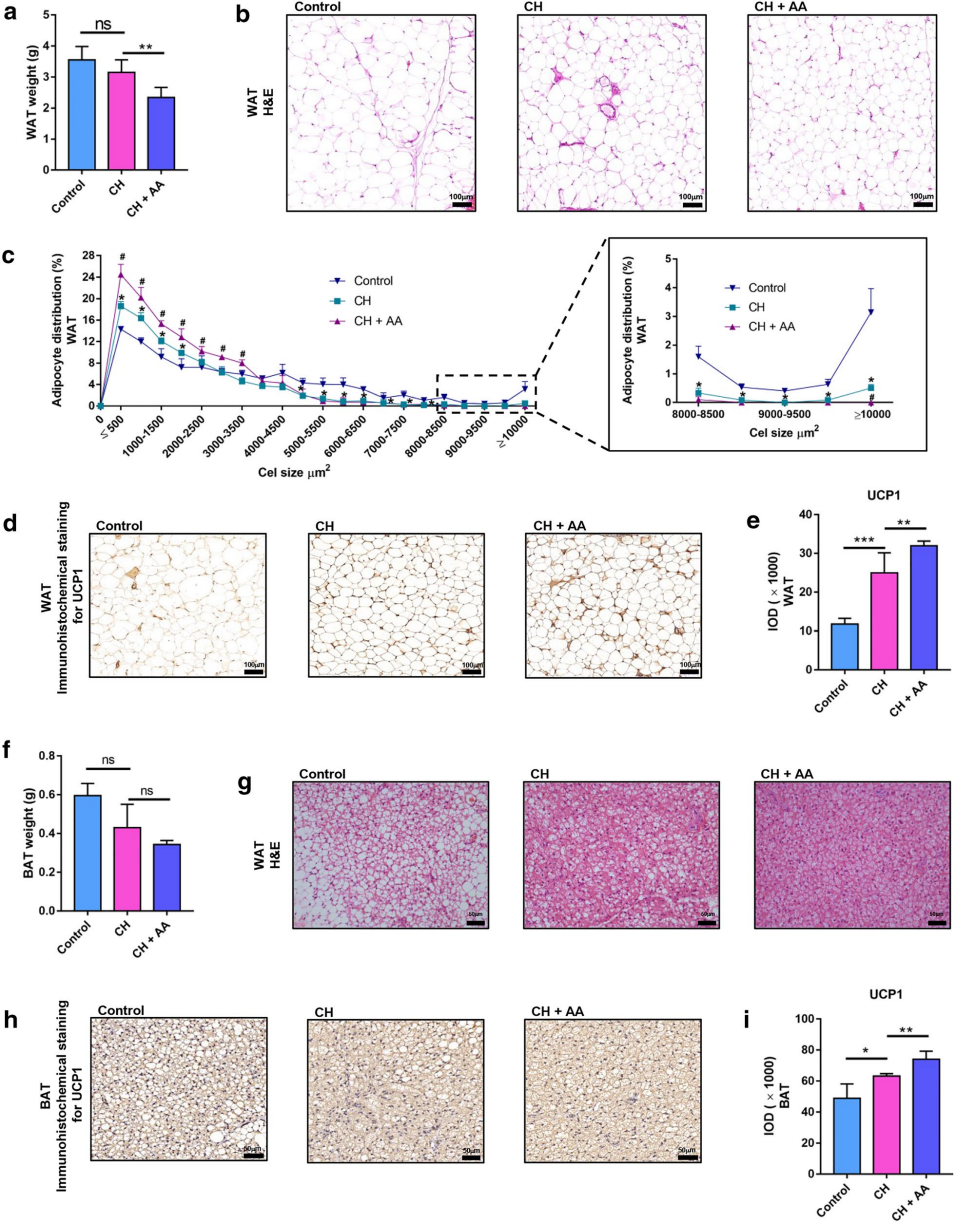
AA promoted browning of WAT and thermogenesis of BAT. **a** White adipose tissue (WAT) weight. H&E staining of WAT (Magnification = 100×; scale bar, 100 μm) (**b**) and distribution of adipocyte size from WAT (**c**). Significance was represented by *P < 0.05 vs. control group and ^#^*P* < 0.05 vs. CH group. Immunohistochemistry of uncoupling protein 1 (UCP1) of WAT (Magnification = 100×; scale bar, 100 μm) (**d**) and the expression of UCP1 in WAT (**e**). **f** Brown adipose tissue (BAT) weight. **g** H&E staining of BAT (Magnification = 200×; scale bar, 50 μm). (h and i) Immunohistochemical staining of UCP1 in BAT (Magnification = 200×; scale bar, 50 μm) (**h**) and the relative expression of UCP1 in BAT (**i**). Control, CH, and CH + AA represent groups intermittently exposed to warm water (32 ± 0.5 °C), cold water (3.5 ± 0.5 °C), and cold water plus AA, respectively. Data were expressed as mean ± standard deviation (*SD*) (n = 6 rats/group). Differences were evaluated by one-way ANOVA with a post hoc Student–Newman–Keuls test (*P < 0.05, **P < 0.01, ***P < 0.001, and ns represents no significant difference)

Mammalian WAT is an energy-storing tissue in the form of lipids. Browning of WAT is an adaptive and reversible response to environmental stimuli such as cold exposure [34]. Therefore, we investigated the effect of AA on WAT browning. The weight of WAT in the CH group was decreased but not significantly versus the control group, whereas AA significantly reduced WAT weight in CH + AA group compared with the CH group (Fig. 2a). We also measured the size distribution of adipocytes of WAT with the help of Image-Pro Plus software. The AA-treated rats had a more increased number of the small adipocytes and a smaller number of the large adipocytes in WAT (Fig. 2b, c). All of these phenotypes are typical features of browning of WAT, a process that is inducible by exposure to cold conditions and an indicator for improved non-shivering thermogenesis [35]. Because UCP1 is currently considered as the only thermogenic protein that is responsible for non-shivering thermogenesis in adipose and is an indicator of WAT browning [34], we further measured the level of UCP1 in WAT. As expected, hypothermic rats showed increased UCP1 in WAT, and treatment with AA significantly increased UCP1 in WAT (Fig. 2d and e). Taken together, AA promoted the browning of WAT in hypothermic rats.

Because BAT acts as the major source of non-shivering thermogenesis via UCP1 upon cold exposure, we then detected the amount of BAT and the level of UCP1 in BAT. Although the weight of BAT did not show statistical change after AA treatment in hypothermic rats, there was still a conspicuous decreasing trend of BAT weight (Fig. 2f). H&E staining of BAT showed that AA treatment significantly decreased the cytoplasmic lipid droplets of BAT (Fig. 2g), indicating the degradation of lipids and increased thermogenesis in BAT. Then we further used immunohistochemistry to investigate the expression of UCP1 in BAT. Consistent with other studies [36, 37], exposure to cold increased the expression of UCP1 in BAT (Fig. 2h, i). After the AA administration, the expression of UCP1 in BAT was significantly increased in hypothermic rats (Fig. 2h, i). Taken together, these results indicated that AA treatment improved the thermogenesis of BAT.

### Gut microbiota is related to the thermogenesis of AA

**Fig. 3:**
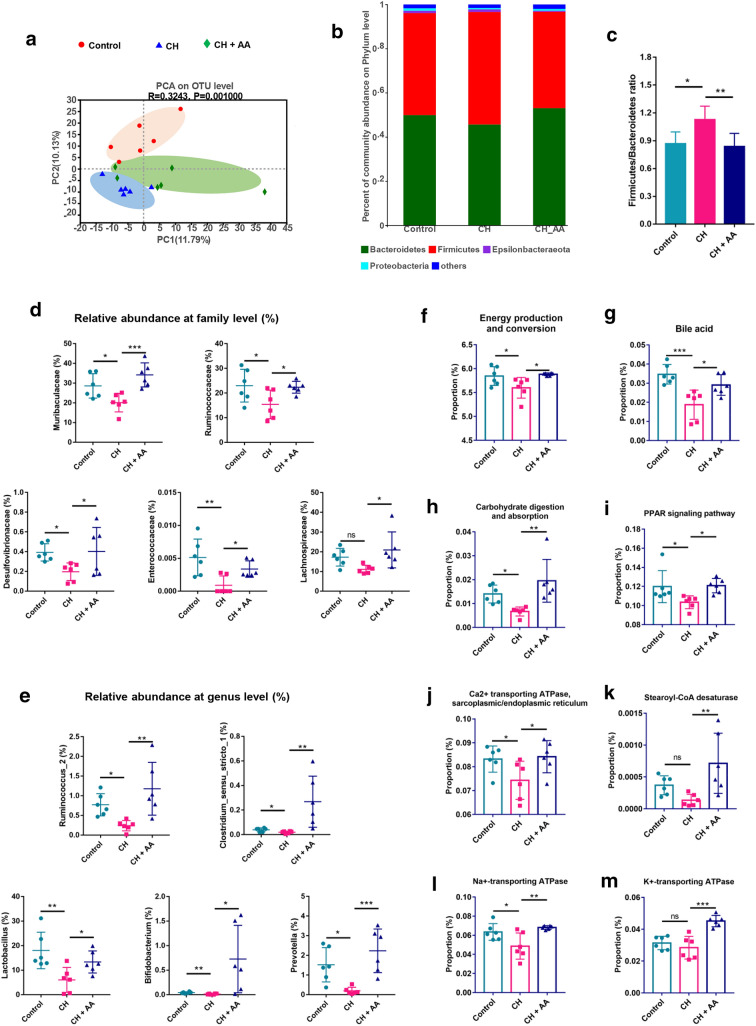
AA reshaped the composition and function of gut microbiota. **a** Principal component analysis (PCA) based on weighted_normalized_unifrac distance metrics of operational taxonomic units (OTUs). **b** Percent abundance of bacteria at the phylum level. **c** *Firmicutes*/*Bacteroidetes* ratio.  The relative abundance of bacteria at the family level (**d**) and the genus level (**e**). The relative abundance of genes involved in energy production and conversion (**f**), and bile acid metabolism (**g**) predicted by COG. The relative abundance of genes involved in carbohydrate digestion and absorption (h) and PPAR signaling pathway (**i**) in the KEGG pathway on level III. (j−m) The gene levels of sarcoplasmic/endoplasmic reticulum Ca^2+^ ATPase (j), stearoyl-CoA desaturase (**k**), Na^+^-transporting ATPase (**l**), and K^+^-transporting ATPase (**m**). Control, CH, and CH + AA represent groups intermittently exposed to warm water (32 ± 0.5 °C), cold water (3.5 ± 0.5 °C), and cold water plus AA, respectively. Data were expressed as mean ± standard deviation (*SD*) (n = 6 rats/group). Differences were evaluated by one-way ANOVA with a post hoc Student–Newman–Keuls test (*P < 0.05, **P < 0.01, and ***P < 0.001, and ns represents no significant difference)

Recent studies have proved that gut microbiota plays an important role in thermogenesis when exposed to cold conditions. We therefore profiled the composition of gut microbiota to investigate the effects of AA on gut microbiota in hypothermic rats. Simpson index, an indicator of alpha diversity, was significantly decreased when the rats were intermittently exposed to severe cold (Additional file 1: Fig. S3A), which was consistent with the previous report [38]. On the contrary, AA treatment increased the Simpson index although there was no statistical significance (Additional file 1: Fig. S3A). PCA, an indicator of beta diversity, showed that there was a remarkable difference between the control group and CH group whereas AA treatment showed a trend to shift toward the control group (Fig. 3a). At the phylum level, the major bacteria were *Bacteroidetes* and *Firmicutes* in all groups (Fig. 3b, Additional file 1: Fig. S3B and C). Intermittent exposure to severe cold increased *Firmicutes*/*Bacteroidetes* ratio whereas AA treatment significantly reversed this ratio (Fig. 3c). The hierarchical clustering heat map showed the major taxa in each group at the family level and genus level (Additional file 1: Fig. S3D and E). To identify statistically significant taxa, the results of LEfSe (Additional file 1: Fig. S4A and B) were subjected to ANOVA. Results showed that, at the family level, the abundances of *Muribaculaceae*, *Ruminococcaceae*, *Desulfovibrionaceae*, *Enterococcaceae*, and *Lachnospiraceae* in the CH group were less than those in the control group. After the treatment of AA, the abundances of all the above-mentioned bacteria were significantly increased (Fig. 3d). At the genus level, the abundances of *Ruminoccoccus*_2, *Clostridium_sensu_stricto*_1, *Lactobacillus*, *Bifidobacterium*, and *Prevotella* were reduced in the CH group compared with the control group. After AA treatment, the abundances of these bacteria were increased in CH + AA group compared with the CH group (Fig. 3e). These data suggested that intermittent exposure to severe cold reshaped the composition of gut microbiota and AA treatment partially restored the disordered gut microbiota community.

The compositional analysis of gut microbiota can answer the question of who exists but cannot answer the question of how the gut microbiota functions. We therefore used PICRUSt to further delve into the functional differences among the three groups. The COG functional classification results showed that 24 functions including energy production and conversion were involved in each group (Additional file 1: Fig. S5). We then quantitatively compared the function of gut microbiota in each group. Noticeably, the function of energy production and conversion was reduced in the CH group whereas AA treatment significantly increased this function (Fig. 3f), which was consistent with our results that AA improved thermogenesis. In addition, AA partially restored the decreased functions of BA metabolism, carbohydrate digestion and absorption, PPAR signaling pathway, all of which play important roles in the regulation of energy metabolism, induced by intermittent exposure to severe cold (Fig. 3g−i). Furthermore, the enzymes directly participating in energy metabolism, including sarcoplasmic/endoplasmic reticulum Ca^2+^ ATPase, stearoyl-CoA desaturase, Na^+^-transporting ATPase, and K^+^-transporting ATPase, were significantly increased by AA administration (Fig. 3j−m). Collectively, these results illustrated that AA not only partially restored the composition of disordered gut microbiota but also the function of the gut microbiota involved in thermogenesis.

## Thermogenesis‐promoting effect of AA is associated with BAs

**Fig. 4:**
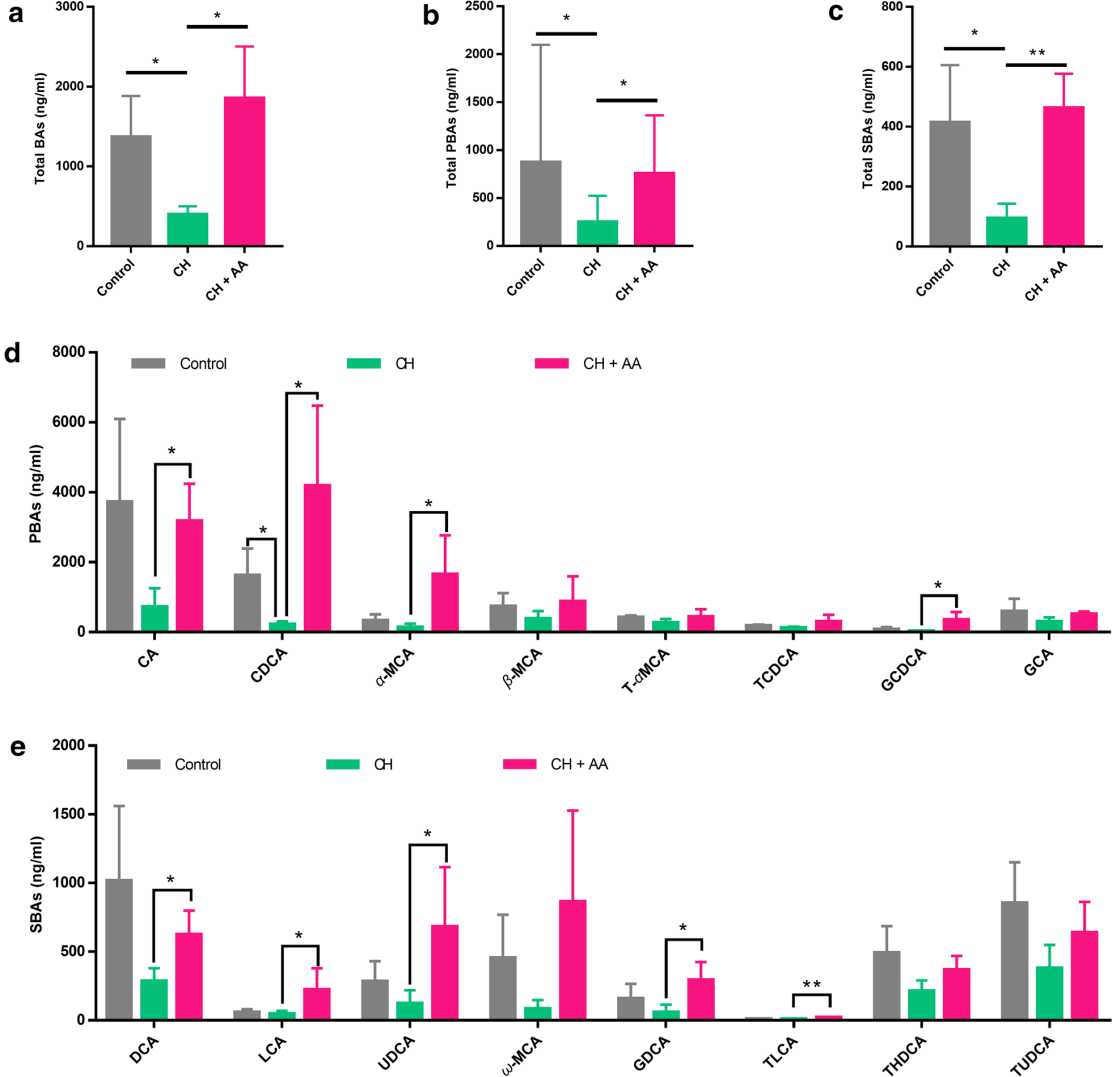
Thermogenesis-promoting effect of AA was associated with BAs. (a−c) The levels of total bile acids (BAs) (**a**), total primary bile acids (PBAs) (**b**), and total secondary bile acids (SBAs) (**c**) in serum.  Specific levels of PBAs (**d**) and SBAs (**e**) in serum. Control, CH, and CH+ AA represent groups intermittently exposed to warm water (32 ± 0.5 °C), cold water (3.5 ± 0.5 °C), and cold water plus AA, respectively. CA, cholic acid; CDCA, chenodeoxycholic acid; *α*-MCA, *α*-muricholic acid; *β*-MCA, *β*-muricholic acid; T-*α*MCA, tauro-*α*-muricholic acid; TCDCA, taurochenodeoxycholic acid; GCDCA, glycoursodeoxycholic acid; GCA, glycocholic acid; DCA, deoxycholic acid; LCA, lithocholic acid; UDCA, ursodeoxycholic acid; *ω*-MCA, *ω*-murichoclic acid; GDCA, glycodeoxycholic acid; TLCA, taurolithocholic acid; THDCA, taurohyodeoxycholic acid; TUDCA, tauroursodeoxycholic acid. Data were expressed as mean ± standard deviation (*SD*) (n = 6 rats/group). Differences were evaluated by one-way ANOVA with a post hoc Student–Newman–Keuls test (*P < 0.05 and **P < 0.01 )

The profound influence of gut microbiota on the host is closely related to the complicated interactions involving a series of host-microbe metabolic axes [39]. BAs, synthesized by the liver and transformed by gut microbiota [40], play important roles in the regulation of energy homeostasis of hosts [41]. To further confirm the PICRUSt result of BA metabolism (Fig. 3g), we used targeted metabolomics to examine the BAs. After intermittent exposure to severe cold, the total BAs, total PBAs, and total SBAs were significantly reduced (Fig. 4a−c). After AA treatment, the levels of total BAs, total PBAs, and total SBAs were recovered (Fig. 4a−c). For the specific BAs, PBAs including CA, chenodeoxycholic acid (CDCA), *α*-muricholic acid (*α*-MCA), and glycochenodeoxycholic acid (GCDCA), as well as SBAs including DCA, lithocholic acid (LCA), ursodeoxycholic acid (UDCA), glycodeoxycholic acid (GDCA), and taurolithocholic acid (TLCA), were significantly increased by AA (Fig. 4d and e). Other BAs such as *β*-muricholic acid (*β*-MCA), *ω*-murichoclic acid (*ω*-MCA), and tauroursodeoxycholic acid (TUDCA) showed a downward trend in the CH group and AA treatment reversed this trend although no statistical significance was observed (Fig. 4d and e). Taken together, the thermogenesis-promoting effect of AA is closely associated with the increase of BAs.

### **Gut microbiota and BAs are responsible for AA to improve hypothermia and increase energy uptake**

**Fig. 5:**
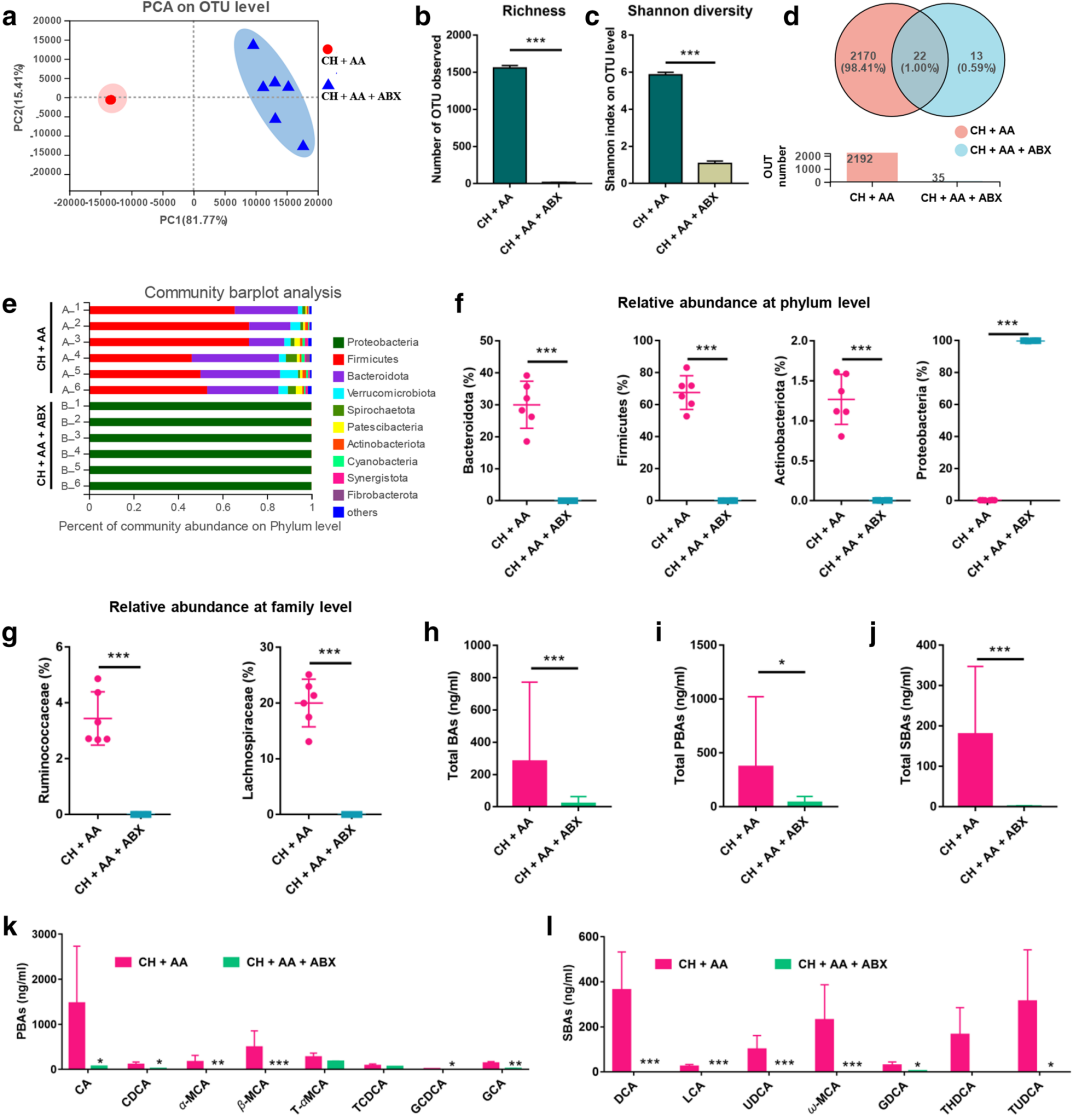
ABX depleted most gut bacteria and decreased BAs. **a** Principal component analysis (PCA) based on weighted_normalized_unifrac distance metrics of operational taxonomic units (OTUs). Observed species (**b**) and Shannon index (**c**) in each group. **d** Existence of OTUs in each group.  The percent of bacteria abundance at the phylum level (**e**) and the typical specific relative abundance of bacteria at the phylum level (**f**) and family level (**g**). The levels of total bile acids (BAs) (**h**), total primary bile acids (PBAs) (**i**), and total secondary bile acids (SBAs) (**j**) in serum. Specific levels of PBAs (**k**) and SBAs (**l**) in serum. CH + AA represents rats intermittently exposed to cold water and fed AA, while CH + AA + ABX represents rats intermittently exposed to cold water and fed AA and antibiotics. CA, cholic acid; CDCA, chenodeoxycholic acid; *α*-MCA, *α*-muricholic acid; *β*-MCA, *β*-muricholic acid; T-*α*MCA, tauro-*α*-muricholic acid; TCDCA, taurochenodeoxycholic acid; GCDCA, glycoursodeoxycholic acid; GCA, glycocholic acid; DCA, deoxycholic acid; LCA, lithocholic acid; UDCA, ursodeoxycholic acid; *ω*-MCA, *ω*-murichoclic acid; GDCA, glycodeoxycholic acid; TLCA, taurolithocholic acid; THDCA, taurohyodeoxycholic acid; TUDCA, tauroursodeoxycholic acid. Data were expressed as mean ± standard deviation (*SD*) (n = 6 rats/group). Differences were evaluated by unpaired two-tailed Student’s *t*-test (*P < 0.05, **P < 0.01, and ***P < 0.001)

In order to further confirm whether the thermogenesis-promoting effect of AA is dependent on the presence of gut microbiota and BAs, we treated hypothermic rats with ABX and then administrated AA for treatment (Additional file 1: Fig. S1B). On the 21st day, we measured the composition of gut microbiota. The PCA and hierarchical clustering tree illustrated that ABX significantly reshaped the gut microbiota composition (Fig. 5a and Additional file 1: Fig. S6A). The observed species and Shannon index were significantly decreased in the CH + AA + ABX group compared with the CH + AA group (Fig. 5b, c, and Additional file 1: Fig. S6B). The Venn diagram analysis showed that only 35 OTUs were existed in the CH + AA + ABX group, while 2192 OTUs were observed in the CH + AA group, corresponding to a 98.40% reduction in gut microbiota by ABX (Fig. 5d). These differences of gut microflora were also reflected in the relative abundance of bacteria at the phylum level (Fig. 5e, f). The relative abundance of bacteria at the family level and genus level also showed a significant decrease of bacteria after ABX treatment (Fig. 5g and Additional file 1: Fig. S6C−F). Taken together, ABX significantly decreased the diversity and abundance of gut bacteria.

Given that BAs are positively correlated with thermogenesis, we assessed the changes in BAs after ABX treatment. As expected, the targeted metabolomics profiling of serum BAs revealed that ABX treatment significantly reduced the concentrations of total BAs, total PBAs, and toral SBAs in the CH + AA + ABX group compared with that in the CH + AA group (Fig. 5h−j). Specifically, the levels of PBAs, including CA, CDCA, *α*-MCA, *β*-MCA, GCDCA, and glycocholic acid (GCA) in the CH + AA + ABX group were significantly decreased compared with that in the CH + AA group (Fig. 5k). And the concentrations of SBAs, including DCA, LCA, UDCA, *ω*-MCA, GDCA, and TUDCA in the CH + AA + ABX group were significantly decreased compared with that in the CH + AA group (Fig. 5l). Taken together, gut microbiota depletion by ABX significantly decreased the concentrations of all BAs.

**Fig. 6:**
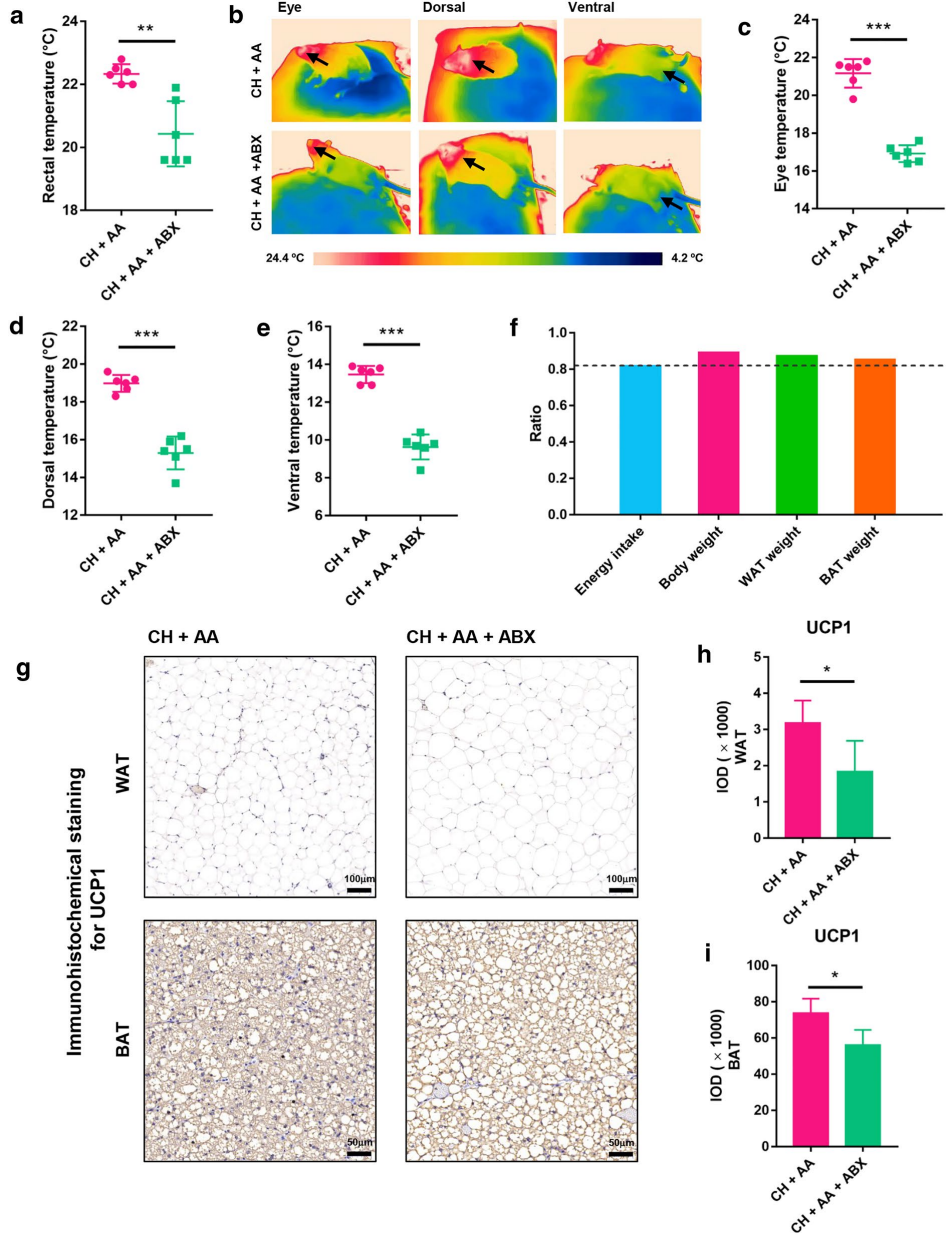
Reduction of gut microbiota and BAs weakened the thermogenesis-promoting effect of AA. **a** Rectal temperature. **b** Representative infrared images. The black arrow points to the temperature measurement location, including the eyes, back, and abdomen. (c–e) Surface body temperature including eye temperature (**c**), dorsal temperature (**d**), and ventral temperature (**e**). (f) The ratio of energy intake, body weight, white adipose tissue (WAT) weight, and brown adipose tissue (BAT) weight of CH + AA + ABX to energy intake, body weight, WAT weight, and BAT weight of CH + AA, respectively. Immunohistochemical staining (**g**) for uncoupling protein 1 (UCP1) in WAT (Magnification = 100×; scale bar, 100 μm) and BAT (Magnification = 200×; scale bar, 50 μm), and the relative expression of UCP1 in WAT (**h**) and BAT (**i**). CH + AA represents rats intermittently exposed to cold water and fed AA, while CH + AA + ABX represents rats intermittently exposed to cold water and fed AA and antibiotics. Data were expressed as mean ± standard deviation (*SD*) (n = 6 rats/group). Differences were evaluated by unpaired two-tailed Student’s *t*-test (*P < 0.05, **P < 0.01, and ***P < 0.001)

We then explored whether the depletion of gut microbiota and reduction of BAs could impair the thermogenesis-promoting effect of AA on hypothermia. After 7 days of AA treatment, we found that rectal temperature was significantly reduced in the CH + AA + ABX group compared with the CH + AA group (Fig. 6a and Fig. S7A). Infrared imaging was used to measure the surface body temperature including eye temperature, as well as ventral and dorsal temperature (Fig. 6b). Results showed that the gut microbiota depletion by ABX significantly reduced the eye temperature, dorsal, and ventral temperature in the CH + AA + ABX group compared with that in the CH + AA group (Fig. 6c−e and Fig. S7B−D). The results indicated that the depletion of gut microbiota and the reduction of BAs reduced the effect of AA on improving hypothermia. To further confirm the effects of gut microbiota and BAs on energy uptake, we measured the food intake, feces amount, food and fecal calories to calculate the total energy intake (Fig. S7E−I). As expected, we found a significant reduction in energy intake in the CH + AA + ABX group compared with the CH + AA group (Fig. S7I). The result confirmed that gut microbiota and BAs are responsible for AA to increase energy uptake. In addition, we also calculated the ratio of energy intake, body weight, WAT, and BAT weight in the CH + AA + ABX group to the CH + AA group. Compared with the ratio of energy intake in the CH + AA + ABX group to the CH + AA group, the ratio of body weight, WAT weight, and BAT weight in the CH + AA + ABX group to the CH + AA group was higher (Fig. 6f). The result suggested that the depletion of gut microbiota and the reduction of BAs reduced the thermogenesis-promoting effect of AA.

### Gut microbiota and BAs are responsible for AA to promote the browning of WAT and thermogenesis of BAT

To further confirm the role of gut microbiota and BAs in the thermogenesis-promoting effect of AA, we measured the size distribution of adipocytes of WAT. ABX treatment increased the number of large adipocytes and decreased the number of small adipocytes in WAT (Fig. S7J and K). We also measured the level of UCP1 in WAT. As expected, ABX treatment significantly decreased UCP1 in WAT in AA-treated rats (Fig. 6g, h). These results collectively indicated that ABX decreased the browning of WAT and non-shivering thermogenesis in AA-treated rats. H&E staining of BAT showed that ABX treatment significantly increased the cytoplasmic lipid droplets of BAT (Fig. S7L), indicative of increased storage of lipids and decreasing of thermogenesis in BAT. Then we further used immunohistochemistry to investigate the expression of UCP1 in BAT. As a result, ABX treatment significantly reduced the level of UCP1 in BAT of the CH + AA + ABX group (Fig. 6g and i), indicative of decreasing of thermogenesis in BAT in ABX-treated rats. Taken together, the results confirmed that gut microbiota and BAs are responsible for AA to promote the browning of WAT and thermogenesis of BAT.

## Discussion

Recent studies have shown that polysaccharides and other types of drugs with low bioavailability can act on gut microbiota to achieve therapeutic effects, therefore, we investigated the effects of AA on gut microbiota. After AA treatment, a remarkable shift of gut microbial structure was observed (Fig. 3a) and an increasing trend of *Firmicutes*/*Bacteroidetes* ratio induced by cold exposure was reduced (Fig. 3c). At the family level, AA treatment enriched the abundance of *Ruminococcaceae*, *Desulfovibrionaceae*, and *Enterococcaceae* (Fig. 3d). Those three bacterial taxa have been reported to be beneficial for energy expenditure and WAT browning [42–44]. At the genus level, AA treatment significantly increased the levels of *Lactobacillus* and *Prevotella* (Fig. 3e). *Lactobacillus* and *Prevotella* were directly associated with the expression of UCP1 in BAT and WAT [45, 46], a direct indicator of thermogenesis of BAT and WAT [47, 48]. These results collectively indicated that AA may promote thermogenesis by modulation of gut microbiota composition.

Among a series of gut microbiota-related metabolites, BAs play important roles in maintaining energy homeostasis [49]. One interesting result is that, after exposure to cold conditions, the contents of most BAs were reduced in our study while the contents of BAs in other studies were increased [22]. This reason can be ascribed to the fact that the rats in other study were exposed to 4 °C air (a condition that can be physiologically adapted to by improving thermogenesis) and the rats in our study were exposed to 4 °C water (a harsh condition that cannot be physiologically adapted to and can induce pathological changes). Nevertheless, an increased level of BAs is an important contributor to non-shivering thermogenesis [22]. Our targeted metabolomic profiling of BAs showed that AA administration increased BAs concentrations, including CA, CDCA, DCA, and LCA (Fig. 4d and e). CA and CDCA, the two most common PBAs, as well as DCA and LCA, the two most common SBAs, can induce the activation of G protein-coupled bile acid receptor 1 (GPBAR1, also known as TGR5) in BAT and thereby lead to increased expression of UCP1 and thermogenesis of BAT [50]. In our study, the expression of UCP1 and the levels of BAs were upregulated by AA treatment (Fig. 4a−e). Taken together, these results indicated that AA reshaped the composition and function of gut microbiota favoring the transformation of BAs, and the increased BAs may contribute to the thermogenesis of AA.

To establish the causative relationship between gut microbiota and the therapeutic effect of AA, we then used ABX to eliminate gut bacteria as much as possible. Consistent with the previous report [51], our 16 S rRNA sequencing results showed that ABX treatment damaged most of the gut bacteria species (Fig. 5b−d). The bacteria that can increase BAs, including *Ruminococcaceae*, *Lachnospiraceae*, and *Lactobacillus* [41, 52], were significantly decreased in CH + AA + ABX group (Fig. 5g and Additional file 1: Fig. S6F). Consistent with these results, our targeted metabolomic profiling of BAs showed that BAs were significantly decreased after ABX treatment (Fig. 5h−l). In addition, the depletion of gut microbiota and the reduction of BAs impaired the therapeutic effect of AA on hypothermia, resulting in the reduced rectal, eye, dorsal, and ventral temperature, decreased energy intake, and a decrease in the levels of UCP1 protein in both BAT and WAT. Taken together, the ABX treatment confirmed that the thermogenesis-promoting effect of AA is dependent on the presence of gut microbiota and the BAs modified by gut microbiota.

For drugs administrated orally, the material base responsible for the therapeutic effects may include (1) prototype drug, (2) compounds transformed from prototype drug by gut microbiota or liver, (3) compounds generated by gut microbiota [17]. Aconite contains alkaloids, flavonoids, polysaccharides, and other compounds [12]. Among all these compounds, alkaloids are regarded as the major bioactive compounds [29]. Studies have demonstrated that alkaloids from Aconite can stimulate thermogenesis via acting on mitochondria directly [13]. Therefore, although we have demonstrated that gut microbiota and gut microbiota-produced BAs are responsible for the thermogenesis-promoting effect of AA, the direct action of Aconite alkaloids on targets such as hosts’ mitochondria still contributes a large part to the therapeutic effects in our study. In addition to alkaloids, polysaccharides and other compounds may be extracted and exist in AA. Many studies have demonstrated that polysaccharides can modulate the composition and function of gut microbiota to regulate energy metabolism [53–56]. Therefore, Aconite polysaccharides may also be responsible for the therapeutic effect of AA. Future studies are needed to investigate the specific compounds responsible for the thermogenesis-promoting effects of AA.

## Conclusions

The present study for the first time demonstrated that AA remodels the composition of gut microbiota and BAs, resulting in an increase of thermogenesis in rats with hypothermia. Specifically, the mechanism involves increasing the relative abundance of gut bacteria such as *Lactobacillus* and *Bifidobacterium* that are associated with regulation of energy metabolism, and the increasing of BAs such as CA, CDCA, DCA, and LCA that can promote the thermogenesis of BAT. In addition, ablation of gut microbiota confirmed that gut microbiota and BAs play a causal role in increasing thermogenesis. These findings regarding the thermogenesis-promoting effects of AA provide a novel mechanistic understanding of the “hot” property of Aconite and even TCM. In addition, these findings might also provide a novel solution for the treatment of thermogenesis-associated diseases such as obesity and type 2 diabetes.

## Supplementary Information

**Additional file 1.** Additional figures and tables.

## Data Availability

The datasets used in this study are available from the corresponding author upon reasonable request.

## Abbreviations

AA: Aqueous extracts of Aconite
ABX: Antibiotic cocktail
ANOVA: One-way analysis of variance
BAs: Bile acids
BAT: Brown adipose tissue
CA: Cholic acid
CDCA: Chenodeoxycholic acid
CH: Cold water-induced hypothermia
COG: Cluster of Orthologous Groups of proteins
DCA: Deoxycholic acid
GCA: Glycocholic acid
GCDCA: Glycochenodeoxycholic acid
GDCA: Glycodeoxycholic acid
GPBAR1: G protein-coupled bile acid receptor 1
H&E: Hematoxylin-eosin
HPLC: High-performance liquid chromatography
KEGG: Kyoto Encyclopedia of Genes and Genomes
KO: KEGG Orthology
LCA: Lithocholic acid
LDA: Linear discriminant analysis
LEfSe: Linear discriminate analysis effect size
OTUs: Operational taxonomic units
PBAs: Primary BAs
PCA: Principal component analysis
PICRUSt: Phylogenetic investigation of communities by reconstruction of unobserved states
SBAs: Secondary BAs
SD: Standard deviation
SPF: Specific-pathogen-free
TCM: Traditional Chinese medicine
TLCA: Taurolithocholic acid
TUDCA: Tauroursodeoxycholic acid
UCP1: Uncoupling protein 1
UDCA: Ursodeoxycholic acid
UPLC-QTOF/MS: Ultra-high-performance liquid chromatography coupled with quadrupole time-of-flight mass spectrometry.
UPLC-QTrap-MS/MS: Ultra-performance liquid chromatography-quadrupole linear ion trap mass spectrometry.
WAT: White adipose tissue
*α*-MCA: *α*-Muricholic acid
*β*-MCA: *β*-Muricholic acid
*ω*-MCA: *ω*-Murichoclic acid
