# Supplementary material for: Aqueous extracts of Aconite promote thermogenesis in rats with hypothermia via regulating gut microbiota and bile acid metabolism
Source: Chin Med. 2021 Mar 19;16:29. doi: 10.1186/s13020-021-00437-y (PMC7980327; doi:10.1186/s13020-021-00437-y)
Supplement: Supplementary file 1 — Additional file 1. Additional figures and tables. [file 13020_2021_437_MOESM1_ESM.docx]

**Supplementary material for**

**Original article**

**Aqueous extracts of Aconite** **promote** **thermogenesis in rats with** **hypothermia via regulating gut microbiota and bile acid** **metabolism**

Juan Liu^a,b^, Yuzhu Tan^a^, Hui Ao^b^, Wuwen Feng^a,b,⁎^, Cheng Peng^a,b,⁎^

^a^*School of Pharmacy, Chengdu University of Traditional Chinese Medicine, Chengdu 611130, China*

^b^*State Key Laboratory of Southwestern Chinese Medicine Resources, Chengdu University of Traditional Chinese Medicine, Chengdu 611130, China*

^⁎^Corresponding authors at: *School of Pharmacy, Chengdu University of Traditional Chinese Medicine, Chengdu 611130, China*.

E-mail addresses: jiaoxiake-1@foxmail.com (Wuwen Feng), pengchengcxy@126.com (Cheng Peng).

**Table S1 Results of the qualitative analysis of AA by UPLC-QTOF/MS.**

| **No.** | **Component name** | **Neutral mass (Da)** | **Observed m/z** | **Mass error (ppm)** | **Observed RT (min)** | **Adducts** |
| --- | --- | --- | --- | --- | --- | --- |
| 1 | 3-Acetylaconitine | 687.32548 | 688.3349 | 3.1 | 3.07 | +H |
| 2 | Aconitine | 645.31491 | 646.3213 | -1.5 | 5.82 | +H |
| 3 | Beiwulian | 647.29418 | 646.2844 | -3.8 | 5.83 | -H |
| 4 | Benzoylaconine | 603.30435 | 602.2962 | -1.4 | 5.92 | -H |
| 5 | Benzoylhypaconine | 573.29378 | 572.2875 | 1.7 | 3.28 | -H |
| 6 | Benzoylmesaconine | 589.47383 | 588.47564 | 1.6 | 3.37 | -H |
| 7 | Carmichaeline | 377.25661 | 378.2641 | 0.5 | 2.43 | +H |
| 8 | Coryneine | 195.12593 | 234.0886 | -2.2 | 1.49 | +K |
| 9 | Delbruine | 465.27265 | 488.2632 | 2.6 | 4.49 | +Na |
| 10 | Delgrandine | 741.27853 | 740.2727 | 2 | 2.97 | -H |
| 11 | Delsemine A | 699.37309 | 722.3641 | 2.5 | 4.02 | +Na |
| 12 | Delsemine B | 699.37309 | 722.3611 | -1.7 | 3.31 | +Na |
| 13 | Deltaline | 507.28322 | 530.2704 | -3.8 | 2.66 | +Na |
| 14 | Demethylcoclaurine | 271.12084 | 270.1129 | -2.4 | 2.52 | -H |
| 15 | Denudatine | 343.25113 | 344.2571 | -3.7 | 4.26 | +H |
| 16 | Deoxyaconitine | 629.32 | 630.3291 | 2.9 | 4.53 | +H |
| 17 | Dictysine | 347.24604 | 348.252 | -3.7 | 2.89 | +H |
| 18 | Fuziline (15-*α*-Hydroxyneoline) | 453.27265 | 454.2793 | -1.4 | 3.18 | +H |
| 19 | Fuzinoside | 416.15299 | 415.1451 | -1.6 | 3.81 | -H |
| 20 | Hokbusine A | 603.30435 | 604.3103 | -2.1 | 5.42 | +H |
| 21 | Hokbusine B | 391.23587 | 390.2271 | -3.7 | 1.81 | -H |
| 22 | Hypaconitine | 615.30435 | 616.3129 | 2 | 3.26 | +H |
| 23 | Jesaconitine | 675.32548 | 674.3159 | -3.4 | 6.03 | -H |
| 24 | Mesaconitine | 631.29926 | 632.3081 | 2.5 | 5.13 | +H |
| 25 | Neojiangyouaconitine | 601.32508 | 624.3153 | 1.6 | 3.56 | +Na |
| 26 | Neoline | 437.27774 | 438.2836 | -3.2 | 3.48 | +H |
| 27 | Salsoline | 193.11028 | 194.1179 | 1.7 | 1.45 | +H |
| 28 | Senbusine A | 423.26209 | 424.2693 | -0.1 | 2.63 | +H |
| 29 | Senbusine B | 423.26209 | 446.2519 | 1.4 | 4.43 | +Na |
| 30 | Songorine | 357.23039 | 356.2222 | -2.7 | 3.96 | -H |
| 31 | Talatisamine | 421.28282 | 444.273 | 2.2 | 4.94 | +Na |

**
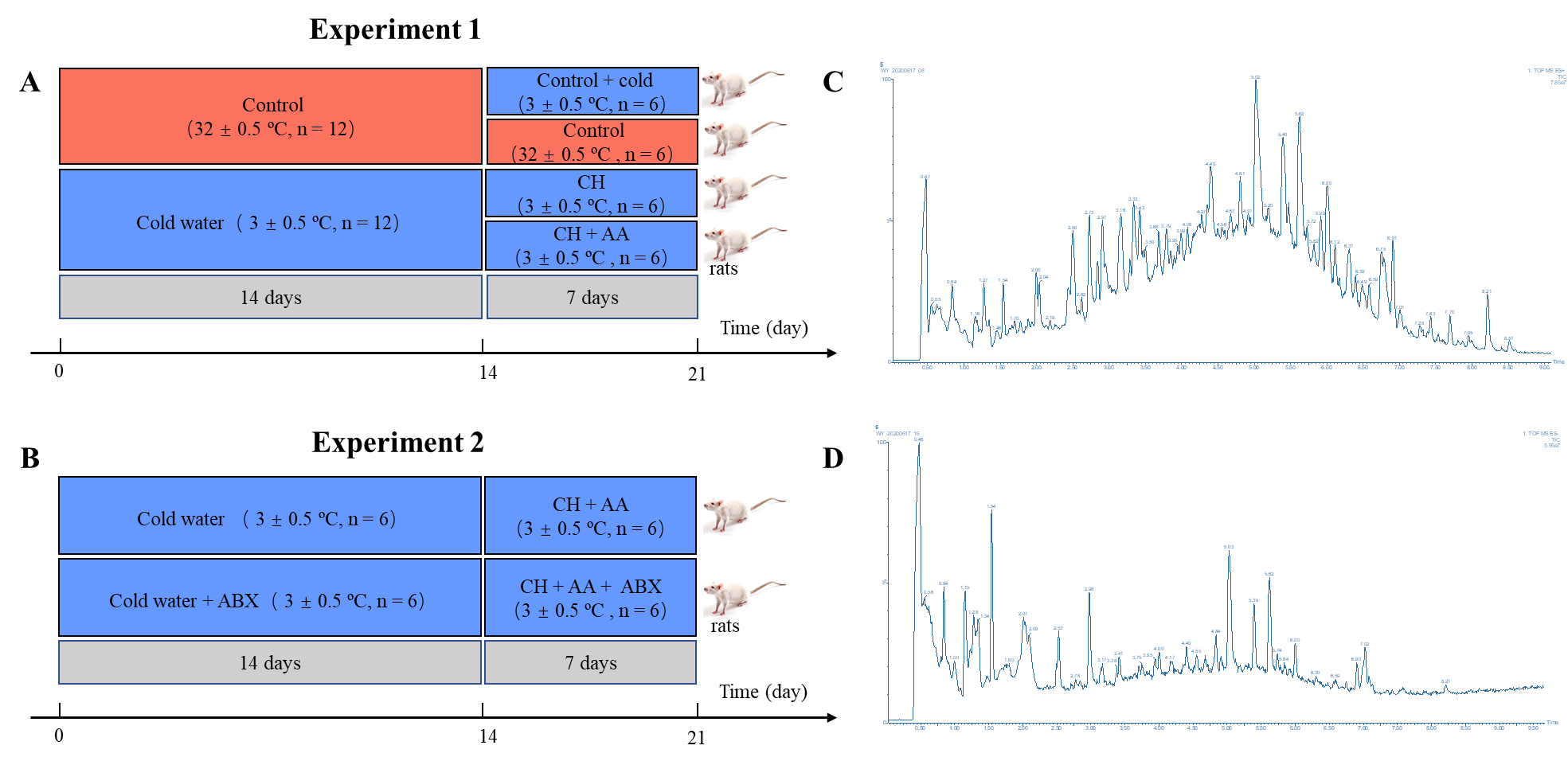
**

**Fig. S1. The design paradigm of the experiment and the total ion chromatogram of AA.**

(A and B) The schematic diagram for experiment 1 (A) and experiment 2 (B).

(C and D) The total ion chromatogram of AA by UPLC-QTOF/MS in ion positive mode (C) and negative ion mode (D).

Experiment 1 was designed to examine the changes in the phenotype, gut microbiota, and bile acids of rats during severe cold exposure and AA treatment. Experiment 2 was used to investigate the role of gut microbiota and bile acids in improving hypothermia after ABX treatment.

**
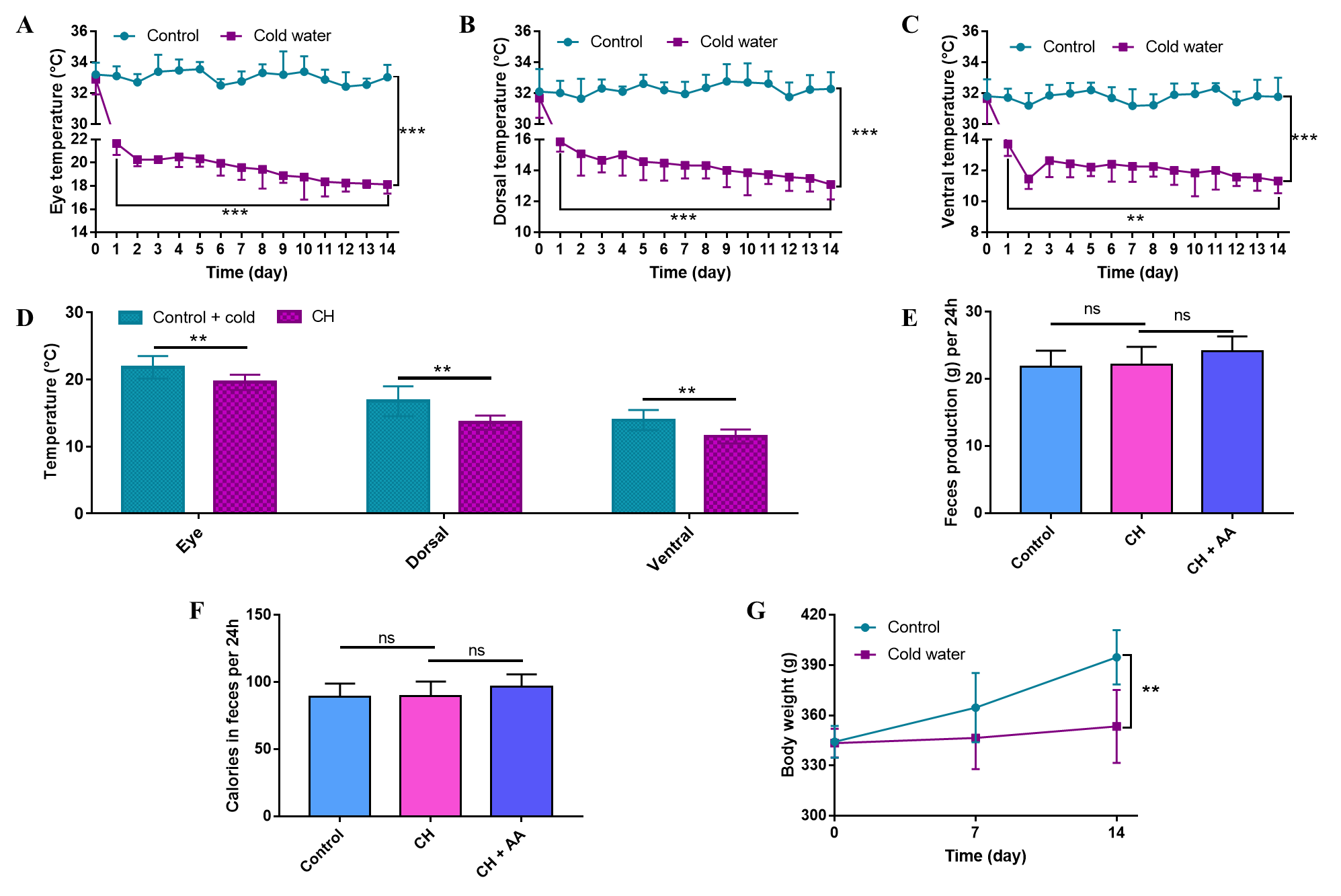
**

**Fig. S2.** Intermittent exposure to severe cold reduced body temperature and energy intake. (A–C) Eye temperature (A), dorsal temperature (B), and ventral temperature (C) from 0 to 14^th^ day. (D) Surface body temperature in the control + cold group and CH group on the14th day. (E and F) Amount of feces excreted (E) and calories in feces (F) per 24 h in the last three days. (G) Body weight on 21^st^ day. Control, CH, and CH + AA represent groups intermittently exposed to warm water (32 ± 0.5 ºC), cold water (3 ± 0.5 ºC), and cold water plus AA, respectively. Data were expressed as mean ± standard deviation (*SD*) (n = 12 rats/group in Fig. S2A–2C and Fig. S2G; n = 6 rats/group in Fig. S2D–2F). Differences were evaluated by unpaired two-tailed Student’s *t*-test or one-way ANOVA with a post hoc Student–Newman–Keuls test (^**^*P* < 0.01, ^***^*P* < 0.001, and ns represents no significant difference).


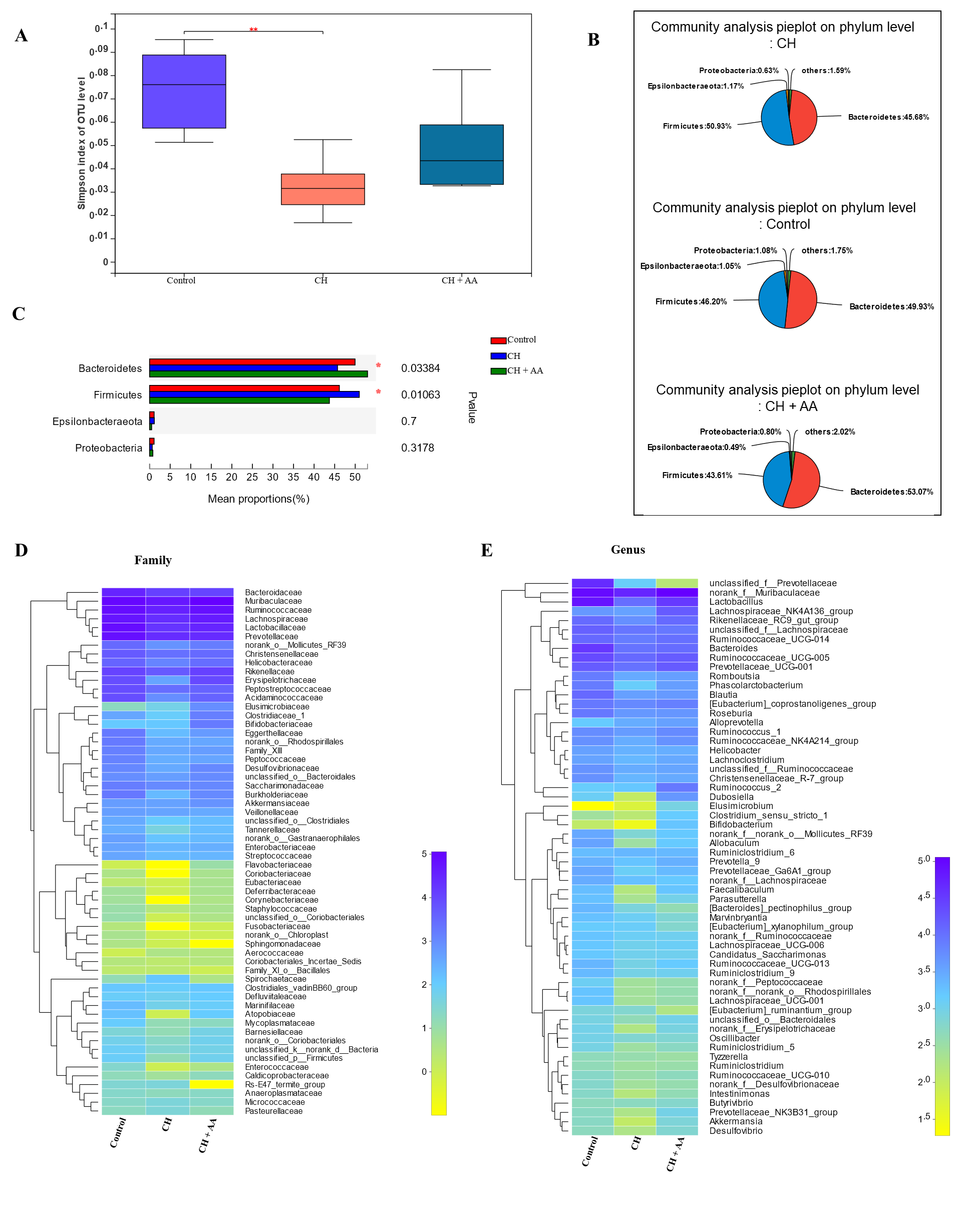


**Fig. S3.** AA reshaped the composition of gut microbiota. (A) Simpson index. (B) Pie plots showing the relative abundance of gut bacteria at the phylum level. (C) Gut bacteria with significant difference among the three groups at the phylum level. (D and E) Heatmap of the representational OTUs at the family level (D) and at the genus level (E). Control, CH, and CH + AA represent groups intermittently exposed to warm water (32 ± 0.5 ºC), cold water (3 ± 0.5 ºC), and cold water plus AA, respectively. Data were expressed as mean ± standard deviation (*SD*) (n = 6 rats/group). Differences were evaluated by using one-way ANOVA with a post hoc Student–Newman–Keuls test (^*^*P* < 0.05 and ^**^*P* < 0.01).


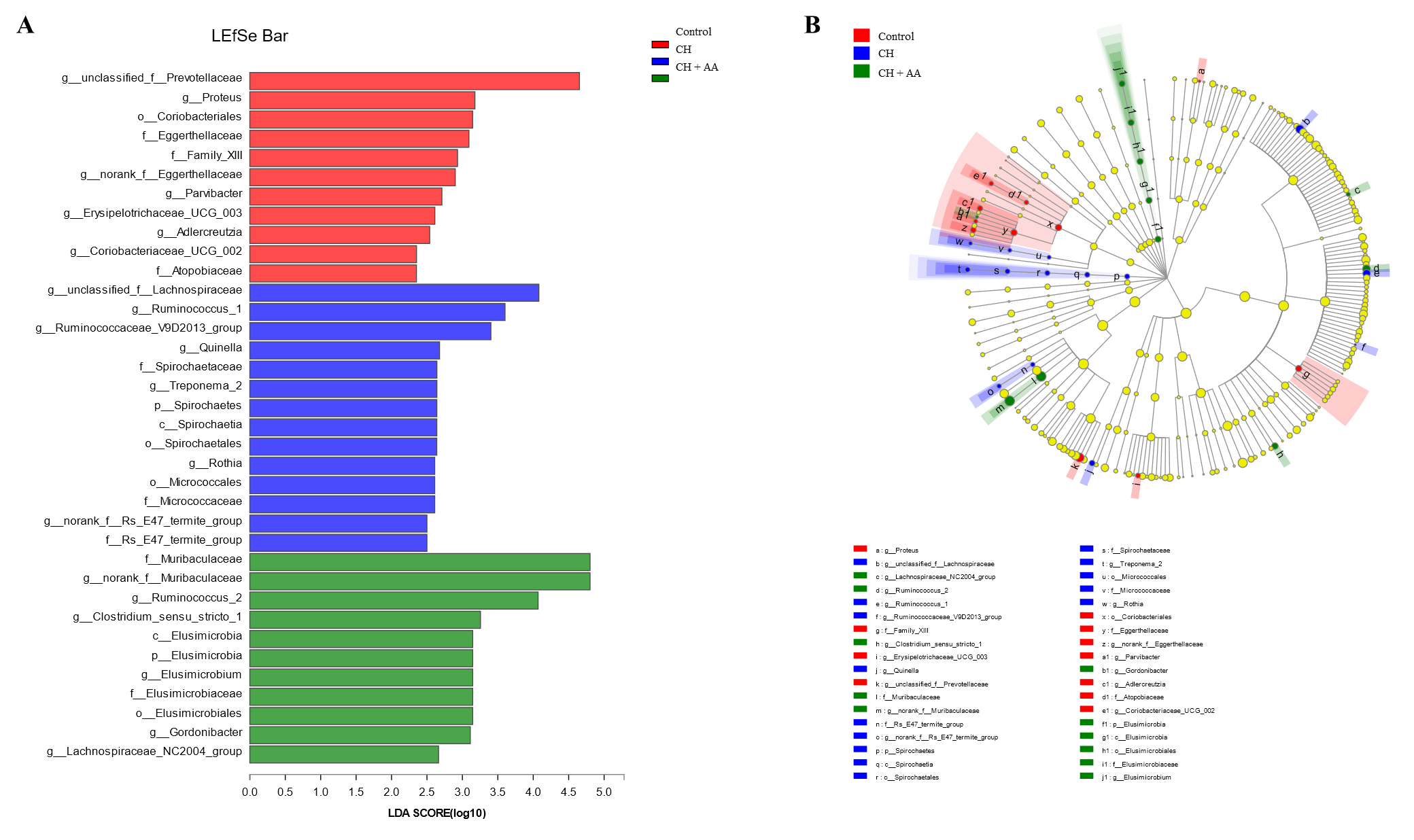


**Fig. S4.** AA reshaped the composition of gut microbiota. Linear discriminant analysis (LDA) scores (A) and cladogram (B) based on linear discriminate analysis effect size (LEfSe) analysis showed the differences of bacterial abundance from the phylum level to the genus level. Control, CH, and CH + AA represent groups intermittently exposed to warm water (32 ± 0.5 ºC), cold water (3 ± 0.5 ºC), and cold water plus AA, respectively. Data were expressed as mean ± standard deviation (*SD*) (n = 6 rats/group).


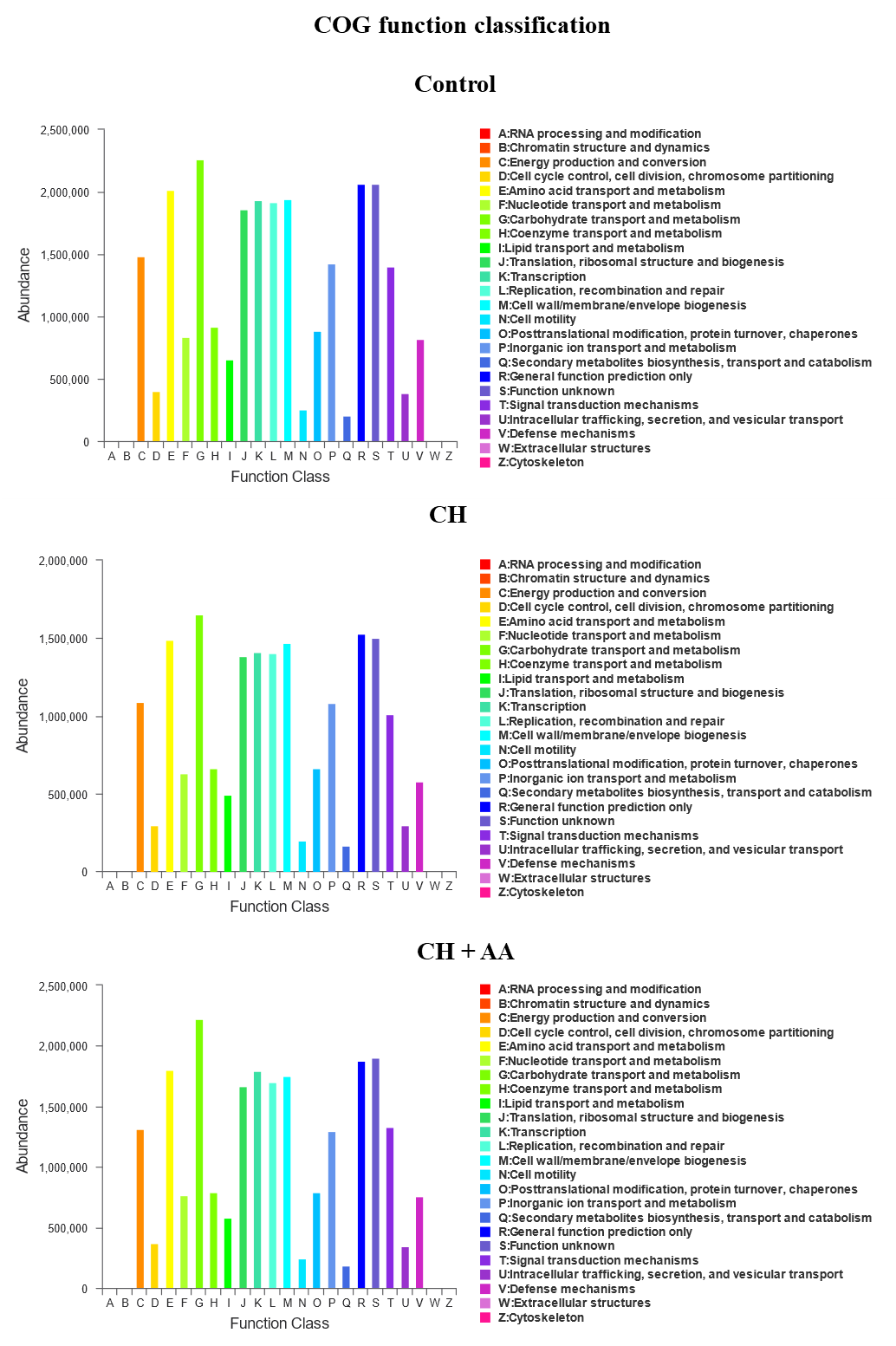


**Fig. S5.** Typical functions of gut microbiota in each group predicted by the Cluster of Orthologous Groups of proteins (COG). Control, CH, and CH + AA represent groups intermittently exposed to warm water (32 ± 0.5 ºC), cold water (3 ± 0.5 ºC), and cold water plus AA, respectively. Data were expressed as mean ± standard deviation (*SD*) (n = 6 rats/group).


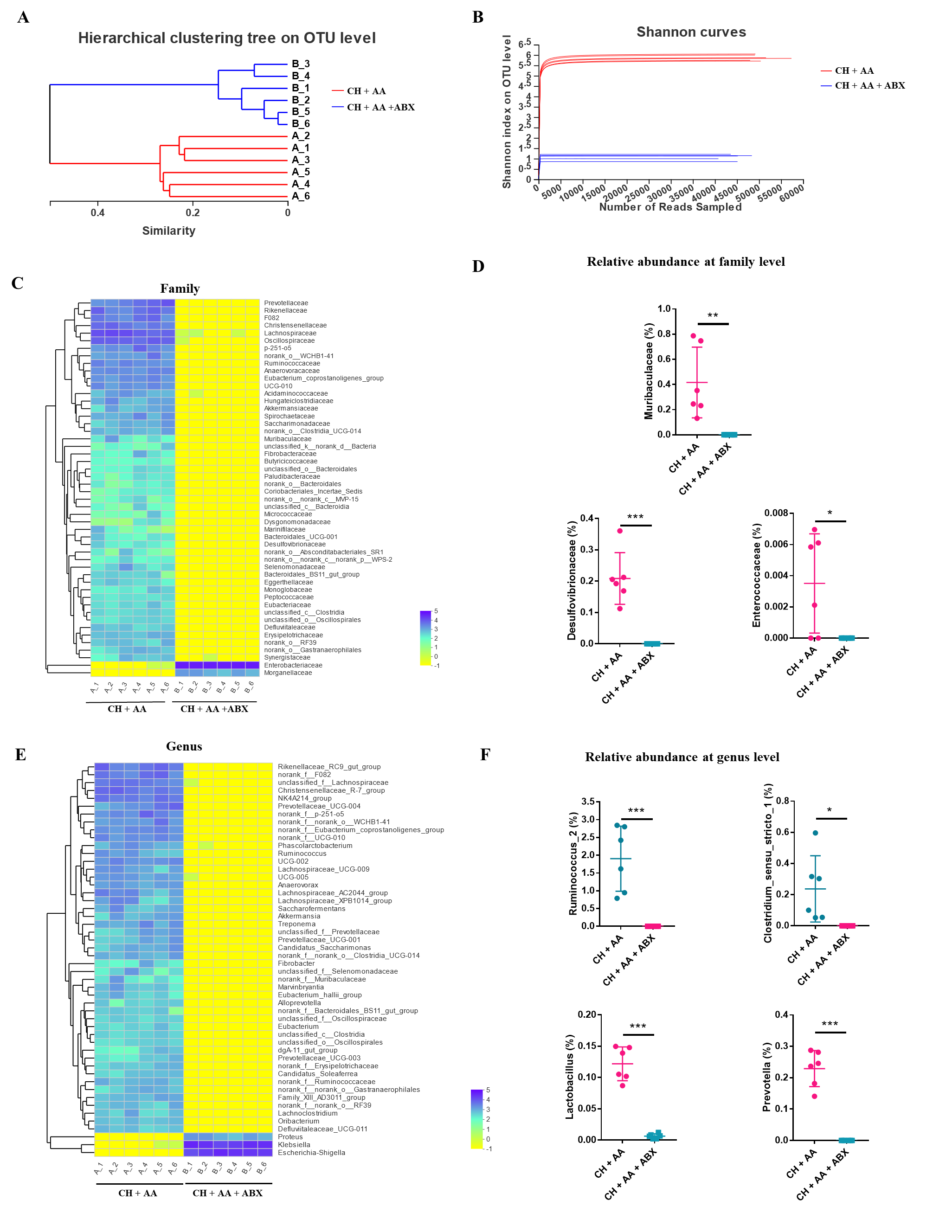


**Fig. S6.** ABX decreased the diversity and abundance of the gut microbiota. (A) The hierarchical clustering tree based on the levels of operational taxonomic units (OUTs). (B) Shannon curves. (C and D) Heatmap of the representational OTUs at the family level (C) and the relative abundance of representative gut bacteria at the family level (D). (E and F) Heatmap of the representational OTUs at the genus level (E) and the relative abundance of representative gut bacteria at the genus level (F). CH + AA represents rats intermittently exposed to cold water and fed AA, while CH + AA +ABX represents rats intermittently exposed to cold water and fed AA and antibiotics. Data were expressed as mean ± standard deviation (*SD*) (n = 6 rats/group). Differences were evaluated by unpaired two-tailed Student’s *t*-test (^*^*P* < 0.05, ^**^*P* < 0.01, and ^***^*P* < 0.001).


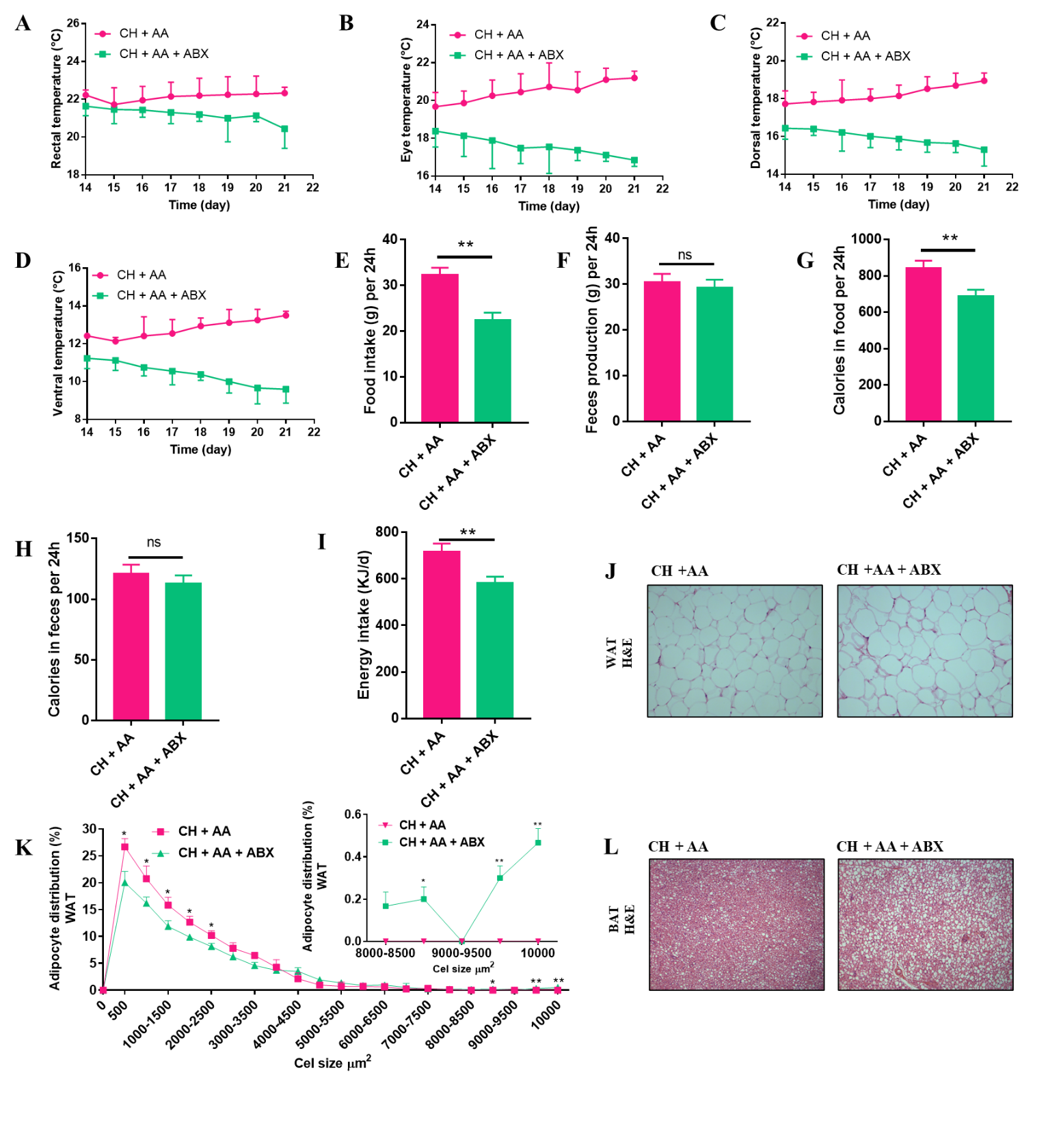


**Fig. S7.** Reduction of gut microbiota and BAs weakened the thermogenesis-promoting effect of AA. (A–D) Rectal temperature (A), eye temperature (B), dorsal temperature (C), and ventral temperature (D) from 14^th^ to 21^st^ day. (E–I) Food intake (E), feces production (F), calories in food (G), calories in feces (H), and energy intake (I). (J–L) H&E staining of white adipose tissue (WAT) (J), the size distribution of adipocyte size in WAT (K), and H&E staining of brown adipose tissue (BAT) (L). CH + AA represents rats intermittently exposed to cold water and fed AA, while CH + AA +ABX represents rats intermittently exposed to cold water and fed AA and antibiotics. Data were expressed as mean ± standard deviation (*SD*) (n = 6 rats/group). Differences were evaluated by unpaired two-tailed Student’s *t*-test (^*^*P* < 0.05, ^**^*P* < 0.01, and ns represents no significant difference).
